# Diagnostic criteria for severe acute malnutrition among infants aged under 6 mo^1^,^2^,^3^

**DOI:** 10.3945/ajcn.116.149815

**Published:** 2017-04-19

**Authors:** Martha Mwangome, Moses Ngari, Greg Fegan, Neema Mturi, Mohammed Shebe, Evasius Bauni, James A Berkley

**Affiliations:** 4 Kenya Medical Research Institute (KEMRI)/Wellcome Trust Research Program, Kilifi, Kenya;; 5 Childhood Acute Illness & Nutrition (CHAIN) Network, Nairobi, Kenya;; 6 Swansea Trials Unit, Swansea University Medical School, Swansea, United Kingdom; and; 7 Centre for Clinical Vaccinology and Tropical Medicine, University of Oxford, Oxford, United Kingdom

**Keywords:** infants under 6 months, MUAC, weight-for-length, SAM, HIV

## Abstract

**Background:** There is an increasing recognition of malnutrition among infants under 6 mo of age (U6M). Current diagnosis criteria use weight-for-length *z* scores (WLZs), but the 2006 WHO standards exclude infants shorter than 45 cm. In older children, midupper arm circumference (MUAC) predicts mortality better than does WLZ. Outcomes may also be influenced by exposure to HIV and size or gestational age at birth. Diagnostic thresholds for WLZ, MUAC, and other indexes have not been fully evaluated against mortality risk among U6M infants.

**Objective:** The aim was to determine the association of anthropometric indexes with risks of inpatient and postdischarge mortality among U6M infants recruited at the time of hospitalization.

**Design:** We analyzed data from a cohort of U6M infants admitted to Kilifi County Hospital (2007–2013), Kenya. The primary outcomes were inpatient death and death during follow-up over 1 y after discharge. We calculated adjusted RRs for inpatient mortality and HRs for postdischarge mortality for different anthropometric measures and thresholds. Discriminatory value was assessed by using receiver operating characteristic curves.

**Results:** A total of 2882 infants were admitted: 140 (4.9%) died in the hospital and 1405 infants were followed up after discharge. Of these, 75 (5.3%) died within 1 y during 1318 child-years of observation. MUAC and weight-for-age *z* score (WAZ) predicted inpatient and postdischarge mortality better than did WLZ (*P* < 0.0001). A single MUAC threshold of <11.0 cm performed similarly to MUAC thresholds that varied with age (all *P* > 0.05) and performed better than WLZ <−3 for both inpatient and postdischarge mortality (both *P* < 0.001). Reported small size at birth did not reduce the risk of death associated with anthropometric indexes.

**Conclusions:** U6M infants at the highest risk of death are best targeted by using MUAC or WAZ. Further research into the effectiveness of potential interventions is required.

## INTRODUCTION

Early infancy represents a period of transition from neonatal life to childhood during which there is rapid growth, neurological and immunologic development, and changes in the mode of feeding. Nutrition programs and surveys have traditionally excluded infants under 6 mo of age (U6M)^8^ because adequate nutrition is assumed to be ensured by breastfeeding (1). However, there is increasing recognition that malnutrition occurs before age 6 mo and is associated with mortality (2–4). An analysis of Demographic and Health Survey data reported that, worldwide, 4.7 million U6M infants have moderate acute malnutrition and 3.8 million have severe acute malnutrition (SAM), diagnosed by using the weight-for-length *z* score (WLZ) (5).

In recognition of this, in 2013, the WHO updated guidelines for the management of SAM to include U6M infants. However, these guidelines are based on “very low quality” evidence (6). The diagnosis of SAM in this age group is based on WLZ, with the use of the same thresholds as for older children. However, the 2006 WHO growth standards exclude infants <45 cm in length (7), and there are concerns about the reliability of anthropometric indexes in early infancy (8, 9). Anthropometric criteria for SAM are traditionally based on the association with the risk of subsequent mortality, ideally in an untreated population (10). In older children, midupper arm circumference (MUAC) is more discriminatory for mortality than is WLZ (11).

There are strikingly few such studies, to our knowledge, that associate anthropometric indexes to mortality among U6M infants. In Ghana, Peru, and India, among infants assessed at the first immunization visit, weight-for-age *z* score (WAZ) predicted mortality better than did WLZ and length-for-age *z* score (LAZ) (12). In the only study to our knowledge to examine MUAC in The Gambia among community children, unadjusted MUAC at the age of first immunizations predicted subsequent infant mortality better than did WLZ, and similar to WAZ (2).

Relations between anthropometry and mortality may be confounded by age, HIV, or low birth weight arising from prematurity or small-for-gestational-age status. Low-birth-weight infants could be classified as malnourished by anthropometry at a single time point despite growing normally. Reflecting these concerns, the WHO and a recent Child Health and Nutrition Research Initiative exercise prioritized establishing diagnostic criteria for SAM among U6M infants as the leading research question in this field (6, 13).

We aimed to determine the association of anthropometric indexes with mortality among a large cohort of Kenyan infants aged 1–6 mo who were at increased risk of short- and long-term mortality because of illness resulting in admission to a hospital (14).

## METHODS

### Study site

The study was conducted in Kilifi, Kenya, where 67% of the population lives on <$1/d (15). The Kilifi County Hospital (KCH) is a level-4 hospital providing primary care and inpatient services. Since 1998, detailed clinical characteristics and anthropometric measurements have been prospectively collected for all pediatric admissions (16). Anthropometric measurements were collected by trained dedicated research team following a standard protocol. The electronic weighing scales (Adam MTB 20) and infantometer (SECA 416) were calibrated and documented weekly.

The Kilifi Health and Demographic Surveillance System (KHDSS) was established in 2002 to record births, pregnancies, migration events, and deaths among ∼260,000 people living in an 891-km^2^ area surrounding the KCH. The population is counted through home visits every 4 mo by trained field assistants. Data are linked to clinical and laboratory data from hospital admissions and were previously used to describe overall pediatric postdischarge mortality among children <13 y old admitted to the KCH between 2004 and 2008 (14). Since January 2007, HIV testing has been systematically offered for all pediatric admissions, in line with the national guidelines for provider-initiated testing and counseling, with the use of 2 rapid antibody tests: Determine (Inverness Medical) and Unigold (Trinity Biotech) (16).

### Study design

A cohort of infants aged 1–6 mo were recruited at the time of hospitalization at KCH and followed up for 1 y. The primary outcomes were death in the hospital or death within 12 mo after discharge. We estimated that >40 postdischarge events provided >80% power to detect minimum HRs of 3.0 for MUAC <11.5 cm and 2.5 for MUAC <12.5 cm compared with MUAC >13.5 cm.

### Study participants

**FIGURE 1:**
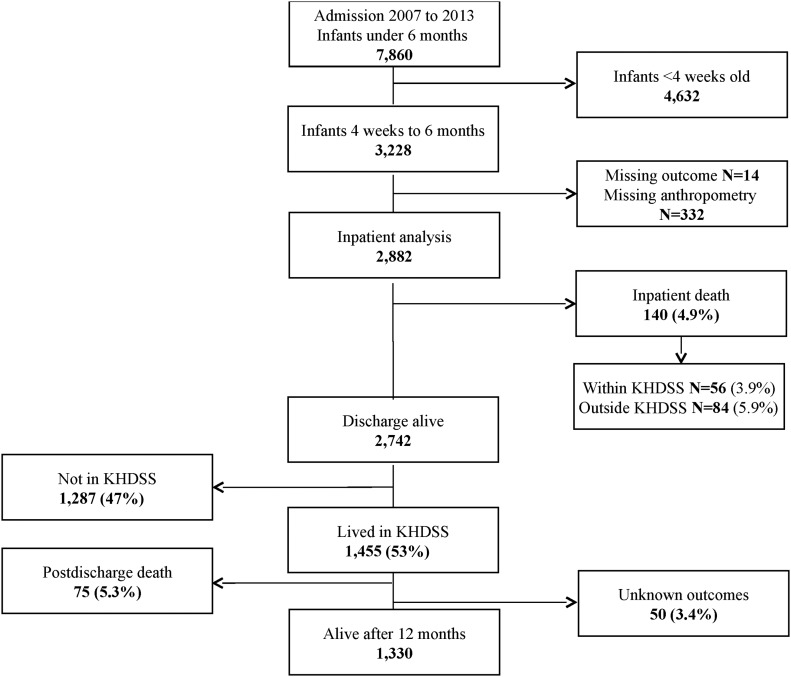
Flowchart of study participants. KHDSS, Kilifi Demographic and Health Surveillance System.

The inpatient analysis included data from all infants aged 4 wk to 6 mo admitted to the KCH between January 2007 and December 2013. The postdischarge analysis included the subset of these infants who were discharged alive, resident within the KHDSS, and followed up from January 2007 to March 2014 (**Figure 1**).

### Statistical analysis

Data were analyzed by using STATA 13.1 (StataCorp). Anthropometric *z* scores were calculated by using the 2006 WHO growth references categorized into a priori groups of normal (≥−1) and mild (−2 to <−1), moderate (−3 to <−2), and severe (<−3) malnutrition. MUAC cutoffs suggested in previous studies (2, 4) were examined. Reference categories of WLZ ≥0 and MUAC ≥13.0 cm were used to define healthy infants, as previously described (2).

To estimate the association between admission anthropometric measurements and inpatient death, RRs were computed by using log-binomial regression models (17). Missing HIV data were analyzed as a separate category because we did not believe they were missing at random.

Postdischarge survival Kaplan-Meier curves were generated and HRs were estimated by using Cox proportional hazards regression models. Time at risk was from discharge to 365 d later, or the date of death or outmigration. We performed a single discharge analysis in which we considered the first admission and discharge for each infant during the period studied. Log-likelihood ratio tests were used to test for interactions. Comparisons of postdischarge survival distributions were performed by using a log-rank test. We adjusted for variables that were potential risk factors or confounders on the basis of previous work (14) and retained all of them in the multivariable models. Goodness-of-fit was assessed by using Akaike’s information criteria. In both inpatient and postdischarge analysis, we created 3 multivariable regression models with the use of the following: *1*) wasting (WLZ; the WHO-recommended anthropometric index for identifying malnutrition in this group) (6), *2*) MUAC, and *3*) underweight (WAZ).

To examine the performance of potential cutoffs for anthropometric indexes, RRs and HRs were calculated for exposure at different cutoffs, including MUAC cutoffs that were fixed or varied by age. We estimated the sensitivity and specificity of different anthropometric thresholds and used the receiver operating characteristic (ROC) curves to describe the discriminatory ability of anthropometric indexes to predict inpatient and postdischarge survival. Fixed-effects meta-analysis was used to obtain pooled estimates of AUCs across each monthly age group. To determine the optimal cutoff for MUAC, the square of distance method, which gives equal weight to both sensitivity and specificity with no ethical, cost, or prevalence constraints, was used, because these data are unavailable (18). The square of distance between the point (0,1) on the upper left-hand corner of the ROC space and any point on the ROC curve was estimated, and the MUAC value with the shortest distance identified and rounded to the nearest 0.5 cm.

### Ethical considerations

Ethical approval was granted by the Kenya National Ethical Review Committee (SCC 2778).

## RESULTS

A total of 2882 infants were admitted and included in the analysis, 1730 (60%) were male (Figure 1), and the median age at admission was 3.0 mo (IQR: 1.7–4.5 mo) (**Supplemental Table 1**). Mean ± SD MUAC and WLZ were 11.9 ± 1.9 cm and −0.76 ± 1.9, respectively (**Table 1**). At admission, 642 (22%) infants were wasted (WLZ <−2), 317 (11%) of whom were severely wasted (WLZ <−3), and 962 (33%) were stunted (LAZ <−2). None of the infants had kwashiorkor (edematous malnutrition); 191 (6.6%) infants had a positive HIV antibody test, 41 (21%) of whom had a WLZ <−3.

**TABLE 1:** Association between anthropometric criteria and inpatient and postdischarge mortality among infants aged 1–6 mo^1^

		Crude (95% CI)		Adjusted^2^ (95% CI)	
	Mean ± SD	RR or HR (95% CI)^3^	AUC (95% CI)	RR or HR (95% CI)^3^	AUC (95% CI)
Inpatient mortality (*n* = 2882)					
WLZ	−0.76 ± 1.9	0.79 (0.72, 0.84)	0.63 (0.58, 0.69)	0.83 (0.76, 0.90)	0.71 (0.66, 0.76)
WAZ	−1.72 ± 1.8	0.66 (0.61, 0.72)	0.74 (0.70, 0.78)	0.68 (0.63, 0.74)	0.76 (0.72, 0.81)
LAZ	−1.46 ± 1.8	0.71 (0.66, 0.77)	0.69 (0.65, 0.74)	0.74 (0.68, 0.80)	0.73 (0.68, 0.78)
MUAC per cm	12.0 ± 1.9	0.67 (0.63, 0.72)	0.75 (0.71, 0.79)	0.69 (0.63, 0.75)	0.77 (0.73, 0.81)
Postdischarge mortality (*n* = 1405)					
WLZ	−0.57 ± 1.8	0.70 (0.62, 0.78)	0.66 (0.59, 0.73)	0.70 (0.62, 0.79)	0.73 (0.67, 0.79)
WAZ	−1.41 ± 1.6	0.57 (0.52, 0.64)	0.78 (0.73, 0.84)	0.56 (0.50, 0.63)	0.80 (0.75, 0.86)
LAZ	−1.21 ± 1.7	0.68 (0.61, 0.75)	0.72 (0.65, 0.78)	0.67 (0.58, 0.78)	0.73 (0.67, 0.79)
MUAC per cm	12.3 ± 1.7	0.61 (0.55, 0.67)	0.77 (0.72, 0.83)	0.58 (0.52, 0.65)	0.81 (0.76, 0.86)

1 An RR or HR of 1 represents no effect; an AUC of 0.5 represents chance and an AUC of 1.0 represents a perfect test. Thus, the best-performing anthropometric index would have the lowest RR or HR and the highest AUC. “RR” indicates an RR per 1-unit *z* score or 1 cm of MUAC; “HR” indicates the HR per 1-unit *z* score or 1 cm of MUAC. LAZ, length-for-age *z* score; MUAC, midupper arm circumference; WAZ, weight-for-age *z* score; WLZ, weight-for-length *z* score.

2 Adjusted for age, sex, HIV, and small size at birth (see Supplemental Table 2).

3 Values are RRs (95% CIs) for “Inpatient mortality” and HRs (95% CIs) for “Postdischarge mortality.”

### Missing data

A total of 346 infants were excluded from the main analysis because of missing data. Of these, 332 were missing data on ≥1 anthropometric measurements: 320 were missing WLZ, 249 were missing LAZ, 106 were missing MUAC, and 63 were missing WAZ, often because infants were too sick to measure at admission. An additional 14 had missing outcomes (Figure 1). Among infants with missing anthropometric measurements, 47 of the 320 (14.7%) infants missing WLZ had a length <45 cm and hence WLZ could not be computed. Adjusted for age and HIV, the RRs of inpatient mortality associated with missing anthropometric indexes were as follows: 3.04 (95% CI: 2.29, 4.04), 5.02 (95% CI: 3.14, 8.00), 3.11 (95% CI: 2.31, 4.19), and 3.56 (95% CI: 2.25, 5.65) for WLZ, WAZ, LAZ, and MUAC, respectively.

### Anthropometry as a predictor of inpatient mortality

Overall, 140 of 2882 (4.9%) infants died during admission. Sixty-nine (49%) deaths occurred within the first 48 h of hospitalization. WLZ, WAZ, LAZ, and MUAC were all associated with inpatient death even after adjusting for confounders (Table 1). The current criteria for diagnosing SAM (WLZ <−3) identified 317 (11%) infants, of whom 40 (12.6%) died. WAZ identified 630 (21.9%) severely underweight infants, of whom 77 (12.2%) died. MUAC <11.0 cm identified 682 (23.7%) infants, of whom 80 (11.7%) died, whereas among the 2200 infants with MUAC ≥11.0 cm, 60 (2.7%) died.

In multivariable analysis, HIV exposure and anthropometric criteria were consistently associated with inpatient mortality (**Supplemental Table 2**). Age was associated with inpatient mortality in the models based on WLZ, WAZ, and LAZ, but not in the MUAC model. Sex was associated with mortality in the LAZ and WAZ models only, and small size at birth (preterm or low birth weight) was associated with mortality in the WLZ model only (Supplemental Table 2).

The adjusted AUCs for WLZ, WAZ, LAZ, and MUAC were 0.71 (95% CI: 0.66, 0.76), 0.76 (95% CI: 0.72, 0.81), 0.73 (95% CI: 0.68, 0.78), and 0.77 (95% CI: 0.73, 0.81), respectively (Table 1, **Supplemental Figure 1**). Compared with WLZ, MUAC and WAZ were better predictors of mortality (both *P* < 0.0001) and LAZ was similar to WLZ (*P* = 0.43).

### Effect of age on MUAC criteria

**FIGURE 2:**
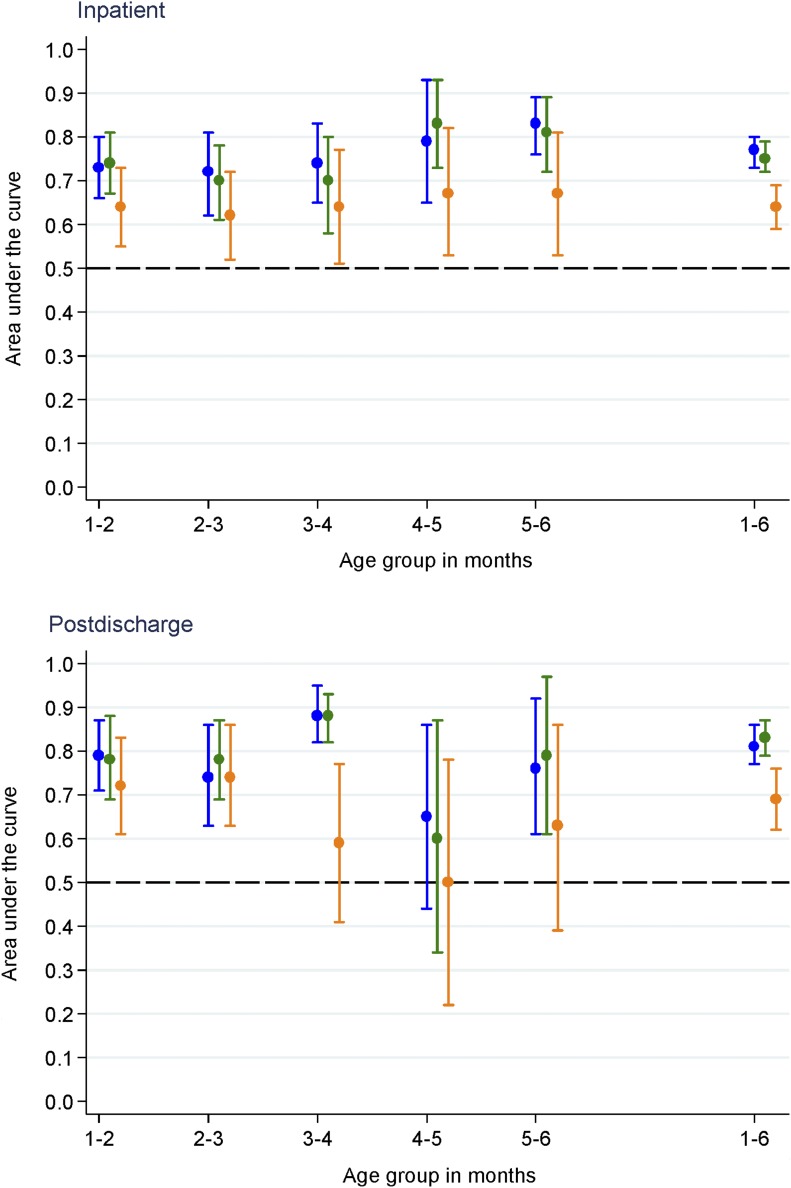
Area under ROC curves (95% CIs) for inpatient (*n* = 2882) and postdischarge (*n* = 1405) mortality associated with MUAC (blue), WAZ (green), and WLZ (orange) by age group. MUAC, midupper arm circumference; ROC, receiver operating characteristic; WAZ, weight-for-age *z* score; WLZ, weight-for-length *z* score.

The effect of age on the interpretation of MUAC was investigated by using a step-wise approach. First, the AUCs for MUAC, WAZ, and WLZ were plotted by month of age. The point estimates of AUC for MUAC and WAZ were consistently above those for WLZ. There was no evidence of heterogeneity between age groups (*I*^2^ = 34.1%, 38.6%, and 0% for MUAC, WAZ, and WLZ, respectively; *P* > 0.05) (**Figure 2**).

**FIGURE 3:**
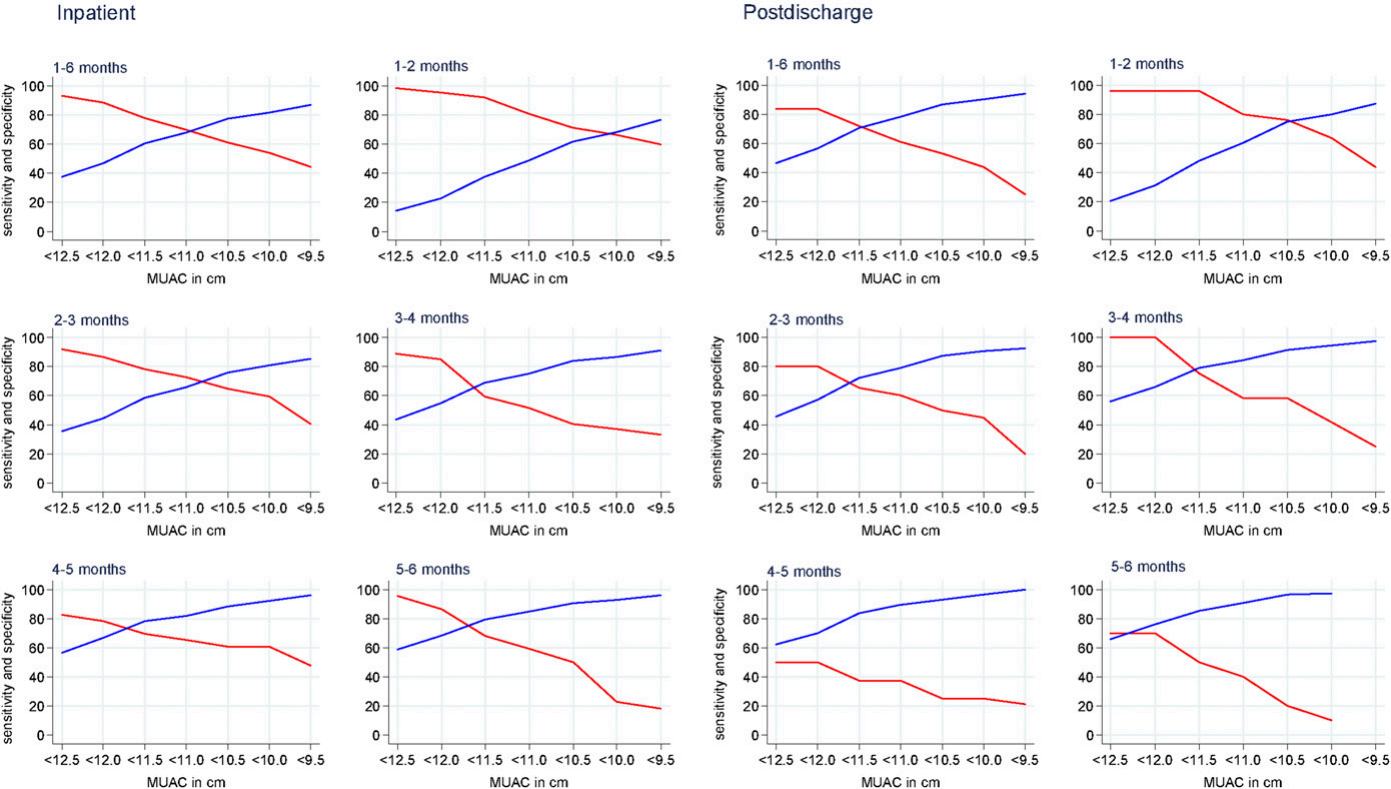
Diagnostic accuracy of different MUAC cutoffs for inpatient (*n* = 2882) and postdischarge (*n* = 1405) mortality overall (1–6 mo) and in different age groups. Red lines indicate sensitivity; blue lines indicate specificity. MUAC, midupper arm circumference.

Second, we investigated optimal cutoffs for MUAC within each age group by using the square distance method and plotted sensitivity and specificity against different MUAC cutoffs. Statistically, the optimal MUAC cutoff was 11.2 cm (rounded down to 11.0 cm). However, the MUAC thresholds differed with age, indicating that age-varying cutoffs should be investigated (**Figure 3**).

To further understand whether single or varied MUAC cutoffs would best suit this age group, we chose <11.0 cm as a reference threshold and tested the diagnostic performance of single and varied MUAC cutoffs against this reference (**Table 2**). For the single criterion between <10.0 and <11.5 cm (Table 2: criteria A–D), we found no difference in the association with the risk of mortality from <11.0 cm; however, differences were found in case load and sensitivity for death. For MUAC criteria that varied by age (AC to AD), the AUC did not differ from <11.0 cm (all *P* > 0.05) (Table 2). The performance of WAZ criteria of <−2 and <−3 was also no better than the use of MUAC <11.0 cm.

**TABLE 2:** Area under ROC curves and sensitivity and specificity of different MUAC and WAZ models for inpatient mortality^1^

Criteria	Cases identified, *n* (%)	Deaths identified, *n* deaths/*n* cases (%)	Sensitivity for death, %	Specificity for death, %	ROC area (95% CI)	*P* value compared with A (Ref)	Infants not identified by criteria (column 1) but identified by WLZ <−3, *n*	Deaths among infants not identified by criteria (column 1) but identified by WLZ <−3, *n*
Inpatient analysis (*n* = 2882)								
A (Ref)	682 (23.7)	80/682 (11.7)	64.3	70.4	0.68 (0.63, 0.72)	—	105	7
B	986 (34.2)	98/986 (9.9)	74.3	62.9	0.69 (0.65, 0.73)	0.40	64	4
C	544 (18.9)	72/544 (13.2)	54.3	79.8	0.67 (0.63, 0.71)	0.63	140	10
D	380 (13.2)	61/380 (16.1)	50.0	84.3	0.66 (0.62, 0.70)	0.27	176	14
Varied MUAC and WAZ criteria for infants compared with Ref								
AC	691 (24.0)	80/691 (11.6)	57.1	77.7	0.67 (0.63, 0.72)	0.87	87	7
BD	487 (16.9)	70/487 (14.4)	50.0	84.8	0.67 (0.63, 0.72)	0.86	128	11
BC	595 (20.7)	76/595 (12.8)	54.3	81.1	0.68 (0.63, 0.72)	0.91	118	9
AD	583 (20.2)	74/583 (12.7)	52.9	81.4	0.67 (0.63, 0.71)	0.74	97	9
WAZ <−2	1050 (36.4)	99/1050 (9.4)	70.7	65.3	0.74 (0.70, 0.79)	0.26	33	3
WAZ <−3	630 (21.9)	77/630 (12.2)	55.0	79.8	0.67 (0.63, 0.72)	0.28	84	5
Postdischarge analysis (*n* = 1405)								
A (Ref)	237 (16.9)	41/237 (17.3)	61.3	78.4	0.70 (0.64, 0.76)	—	47	3
B	373 (26.6)	49/373 (13.1)	72.0	70.7	0.71 (0.65, 0.76)	0.78	29	1
C	180 (12.8)	37/180 (20.6)	53.3	87.0	0.69 (0.64, 0.75)	0.61	63	4
D	111 (7.9)	21/111 (18.9)	44.0	90.2	0.61 (0.55, 0.66)	0.0003	79	8
Varied MUAC and WAZ criteria for infants compared with Ref								
AC	244 (17.4)	41/244 (16.8)	55.4	84.8	0.70 (0.64, 0.76)	0.49	38	2
BD	157 (11.2)	26/157 (16.6)	35.1	90.2	0.63 (0.57, 0.68)	0.005	55	5
BC	203 (14.5)	37/203 (18.2)	50.0	87.5	0.69 (0.63, 0.75)	0.88	51	3
AD	198 (14.1)	30/198 (15.2)	40.5	87.4	0.64 (0.58, 0.70)	0.07	38	2
WAZ <−2	410 (29)	54/410 (13)	72.0	73.2	0.73 (0.67, 0.78)	0.53	18	1
WAZ <−3	219(16)	38/219 (17)	0.51	0.86	0.69 (0.63, 0.74)	0.91	41	2

1 Criteria: A, <11 cm; B, <11.5 cm; C, ≤10.5 cm; D, <10.0 cm; AC, ≤10.5 cm if <3 mo and <11.5 cm if ≥3 mo; BD, <10.0 if <3 mo and <11.0 cm if ≥3 mo; BC, ≤10.5 cm if <3 mo and <11.0 cm if ≥3 mo; AD, ≤10.0 if <3 mo and <11.5 cm if ≥3 mo. MUAC, midupper arm circumference; Ref, reference; ROC, receiver operating characteristic; WAZ, weight-for-age *z* score; WLZ, weight-for-length *z* score.

### Postdischarge mortality

Of the 2742 infants discharged alive, 1455 (50%) lived within the KHDSS and were followed up for 12 mo after discharge (Figure 1). Fifty (3.4%) infants had unknown outcomes; hence, 1405 were included in the final analysis and 851 (61%) of these were male (**Supplemental Table 3**).

During 1318 child-years of observation, 75 (5.3%) infants died, and there was a mortality rate of 57 (95% CI: 45–71) per 1000 child-years of observation. The median time to death was 91 d (IQR: 40, 165 d). A total of 33 of 75 (44%) and 53 of 75 (71%) deaths occurred during the first 3 and 6 mo of follow-up.

In multivariable analysis, HIV exposure and anthropometric criteria were associated with postdischarge mortality (Supplemental Table 2) but not size at birth. The adjusted HRs for postdischarge mortality were 0.70 (95% CI: 0.62, 0.79) for WLZ, 0.56 (95% CI: 0.50, 0.63) for WAZ, 0.67 (95% CI: 0.58, 0.78) for LAZ, and 0.58 (95% CI: 0.52, 0.65) for MUAC (Table 1).

The adjusted AUCs from the anthropometric classification are given in Table 1. Compared with WLZ, MUAC and WAZ were better predictors of mortality (both *P* < 0.001), but LAZ was similar (*P* = 0.93) (Supplemental Figure 1).

The effect of age on the interpretation of the association of MUAC with postdischarge mortality was investigated by first plotting AUCs for MUAC, WAZ, and WLZ by age groups. Within each age group, the point estimate AUCs for MUAC and WAZ were consistently above those for WLZ (Figure 2). There was no evidence of heterogeneity between age group (*I*^2^ = 54.1%, 53%, and 51% for MUAC, WAZ, and WLZ, respectively; *P* > 0.05). Second, by using the square of distance method, the statistically optimal MUAC threshold for all infants was <11.5 cm but varied between age groups (Figure 3).

We tested the performance of the MUAC cutoff of <11.0 cm against other single and varied MUAC cutoffs (Table 2). For the single criterion between <10.0 and <11.5 cm (A–D) we found no differences in association with the risk of mortality from the reference, except for the cutoff of <10.0 cm, which had a significantly lower AUC (*P* = 0.003) than the reference. In all of the groups, differences in case load and sensitivity for death were noted. For the MUAC cutoffs that varied with age, the AUCs did not differ from the single blanket MUAC cutoff of <11.0 cm (*P* > 0.05) (Table 2). WAZ thresholds were not superior to MUAC <11.0 cm (Table 2).

## DISCUSSION

Malnutrition is common and is strongly associated with the risk of inpatient and postdischarge mortality among infants aged 1–6 mo admitted to the hospital in Kenya. Overall, MUAC and WAZ were the strongest predictors of inpatient and postdischarge mortality and should be used for diagnosis and guiding treatment. Within this age group, MUAC thresholds that varied with age did not improve discriminatory value for mortality over a single MUAC criterion of <11.0 cm. The current diagnostic criterion for SAM, WLZ, performed poorly, resulting in missed opportunities for intervention.

### Assessment of malnutrition

The key question that this study addressed is how best to identify malnutrition by anthropometric measures at a single time point in sick infants. It is possible that a single anthropometric assessment may misclassify infants who are low birth weight, either due to prematurity or who were born small-for-gestational age. Such infants may be growing normally, without excess mortality risk. Small size at birth was reported in 7% of the infants in this study. However, for assessment by any anthropometric index, small size at birth did not reduce the elevated risk of death. This suggests that infants identified as malnourished who were born small-for-gestational age should be equally included in interventions.

Another important question is the effect of age on the discriminatory value of anthropometry. Younger infants typically have higher mortality risks, irrespective of anthropometry, and it is known that MUAC is likely to identify younger and shorter infants as being malnourished (11). When we explored the effect of age on the discriminatory value of anthropometric indexes, the effect of WLZ on mortality was confounded by age, WAZ was confounded by age and sex, whereas MUAC was not confounded by any of these factors (Supplemental Table 2). The finding that the association between MUAC and mortality was not affected by age was also shown in earlier studies in older children (19–21). When stratified by age in months, the overall AUCs for MUAC and WAZ were consistently above those for WLZ for both inpatient and postdischarge mortality (Figure 2). This finding is consistent with reports from community U6M infants (2, 12) and children aged 6–59 mo (22–24), in whom MUAC better predicts mortality.

It was clear that a continuous measure of MUAC was effective in identifying infants at a higher risk of mortality. However, in older children, a single cutoff for MUAC is used and we explored different cutoffs that could be applied for diagnosis and intervention among U6M infants. When all U6M infants were considered, an MUAC threshold of <11.0 cm was statistically optimal for the best trade-off between sensitivity and specificity for inpatient mortality, whereas <11.5 cm was statistically optimal for postdischarge mortality. Importantly, the diagnostic MUAC criterion of <11.5 cm, although found to be optimal for postdischarge mortality, did not perform better at discriminating both inpatient and postdischarge mortality when compared with MUAC <11.0 cm. An MUAC cutoff of <11.0 cm would identify 24% of hospitalized infants aged 1–6 mo at risk of inpatient mortality with a sensitivity of 70% and specificity of 68%. For WAZ, <−3 was less sensitive (55%) but more specific (80%) (Table 2). Expanding the MUAC criteria would increase the case load but trade specificity for sensitivity, without corresponding improvements in discriminating infants at a higher risk of mortality.

The population studied comprised sick infants requiring hospitalization. This group was selected because of their elevated risk of mortality, both in the hospital and after discharge, and hence their need for targeted interventions. The population studied differs from community cohorts of healthy children. A previous study in healthy infants in the community (2) equally found that MUAC <11.0 cm showed a better association with mortality than did WLZ. A previous clinical trial that enrolled HIV-negative infants only on the basis of an MUAC <11.0 cm when stabilized after being admitted to 4 rural and urban Kenyan hospitals (4) reported an exceptionally high risk (24.5%) of mortality within the next 12 mo. Collectively, these studies suggest that, among U6M infants, statistically derived MUAC cutoffs are similar in different scenarios.

Ideally, an appropriate anthropometric cutoff for practice would be selected on the basis of which interventions are being applied and their costs and effectiveness. However, because these data are currently lacking because current guidelines have not been evaluated, new approaches are being developed (25). To treat acute malnutrition in U6M infants, the WHO currently recommends re-establishing exclusive breastfeeding (6). However, most U6M infants who are admitted to the hospital because of an acute illness may benefit from exclusive breastfeeding counseling, so this is not a malnutrition-specific intervention. Successfully re-establishing exclusive breastfeeding in an inpatient setting is a labor-intensive, and a time- and resource-consuming, activity (3, 26, 27). Furthermore, for those who are too sick or unable to breastfeed for another reason, dilute Formula 100 or formula milk may be needed (6). The proposed cutoffs can be used to identify infants for whom potentially scarce resources can be prioritized.

### Mortality

Overall, within this cohort there was a ratio of ∼1:1 of inpatient and postdischarge percentage mortality. Other studies have reported a similar ratio within cohorts of severely malnourished children aged 0–59 mo: in Bangladesh, 8.6% died as inpatients and 8.7% died within 3 mo after discharge (28); in Malawi, 14.2% died in the hospital and 16.7% of children discharged died within 250 d (29).

HIV exposure was found in 7% of admissions and was associated with a 3-fold increased risk of mortality, which is similar to the elevated risk reported in older children with malnutrition in Africa (30). However, the majority of the HIV-exposed infants are unlikely to be HIV infected (31). However, exposure status may indicate infection risks in the household, maternal physical and mental health, poverty, and the ability of the mother to provide care. Some infants had missing HIV results; however, this was not associated with an increased risk of mortality.

### Strength and limitations

To our knowledge, this is the first description of mortality in relation to nutritional status among U6M infants admitted to the hospital and after discharge. The major strengths are the large size of the study, the enrollment of infants irrespective of their baseline anthropometric classification, the systematically collected data set, and the duration of follow-up. Limitations are that we were unable to document postdischarge catch-up growth, uptake of HIV services, postdischarge deaths outside of the KHDSS area, or causes of postdischarge mortality.

### Conclusions

Among hospitalized infants aged 1–6 mo, malnutrition is common and is associated with inpatient and postdischarge mortality. MUAC and WAZ are better predictors of mortality than WLZ, as was previously found in community children in The Gambia (2), and these should be used to diagnose acute malnutrition and guide interventions. Further research into the effectiveness and cost-effectiveness of potential interventions in hospitals and subsequently in the community applied with the use of these criteria should be prioritized.

## Footnotes

8 Abbreviations used: KCH, Kilifi County Hospital; KHDSS, Kilifi Demographic and Health Surveillance System; LAZ, length-for-age *z* score; MUAC, midupper arm circumference; ROC, receiver operating characteristic; SAM, severe acute malnutrition; U6M, under 6 mo of age; WAZ, weight-for-age *z* score; WLZ, weight-for-length *z* score.
